# Prostaglandin E2-induced colonic secretion in patients with and without colorectal neoplasia

**DOI:** 10.1186/1471-230X-10-9

**Published:** 2010-01-26

**Authors:** Nicolai Kaltoft, Maria C Tilotta, Anne-Barbara Witte, Philip S Osbak, Steen S Poulsen, Niels Bindslev, Mark B Hansen

**Affiliations:** 1 Department of Surgery K, Bispebjerg Hospital, University of Copenhagen, Copenhagen, Denmark; 2 Department of Gastroenterology and Hepatology, Karolinska University Hospital, Stockholm, Sweden; 3 Department of Anatomy B, Panum Institute, University of Copenhagen, Copenhagen, Denmark; 4 Department of Biomedical Sciences, Panum Institute, University of Copenhagen, Copenhagen, Denmark; 5 AstraZeneca, Research & Development, Mölndal, Sweden

## Abstract

**Background:**

The pathogenesis for colorectal cancer remains unresolved. A growing body of evidence suggests a direct correlation between cyclooxygenase enzyme expression, prostaglandin E_2 _metabolism and neoplastic development. Thus further understanding of the regulation of epithelial functions by prostaglandin E_2 _is needed. We hypothesized that patients with colonic neoplasia have altered colonic epithelial ion transport and express functionally different prostanoid receptor levels in this respect.

**Methods:**

Patients referred for colonoscopy were included and grouped into patients with and without colorectal neoplasia. Patients without endoscopic findings of neoplasia served as controls. Biopsy specimens were obtained from normally appearing mucosa in the sigmoid part of colon. Biopsies were mounted in miniaturized modified Ussing air-suction chambers. Indomethacin (10 μM), various stimulators and inhibitors of prostanoid receptors and ion transport were subsequently added to the chamber solutions. Electrogenic ion transport parameters (short circuit current and slope conductance) were recorded. Tissue pathology and tissue damage before and after experiments was assessed by histology.

**Results:**

Baseline short circuit current and slope conductance did not differ between the two groups. Patients with neoplasia were significantly more sensitive to indomethacin with a decrease in short circuit current of 15.1 ± 2.6 μA·cm^-2 ^compared to controls, who showed a decrease of 10.5 ± 2.1 μA·cm^-2 ^(p = 0.027). Stimulation or inhibition with theophylline, ouabain, bumetanide, forskolin or the EP receptor agonists prostaglandin E_2_, butaprost, sulprostone and prostaglandin E_1 _(OH) did not differ significantly between the two groups. Histology was with normal findings in both groups.

**Conclusions:**

Epithelial electrogenic transport is more sensitive to indomethacin in normal colonic mucosa from patients with previous or present colorectal neoplasia compared to colonic mucosa from control patients. Stimulated epithelial electrogenic transport through individual prostanoid subtype receptors EP1, EP2, EP3, and EP4 is not significantly different between neoplasia diseased patients and controls. This indicates that increased indomethacin-sensitive mechanisms in colonic mucosa from neoplasia diseased patients are not related to differences in functional expression of EP receptor subtypes.

## Background

Colorectal cancer (CRC) is the third most common type of cancer and the second leading cause of death among cancers in the Western world [1]. Therapy is usually through surgery, which in severe cases is followed by chemotherapy [2]. There is a need for additional medical therapy and prevention of CRC, which necessitates further insight into the presently poorly understood mechanisms of colorectal mucosal defence, repair and carcinogenesis. In particular, the mechanisms and signal pathways of pre-neoplastic colorectal epithelial cells are of special interest as these could be target for pharmacotherapy in the prevention of colorectal neoplasia (CRN) and CRC.

**Figure 1:**
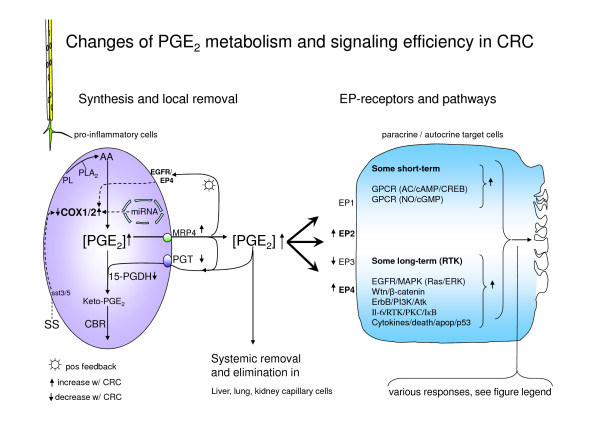
**The synthesis, control of tissue level and signalling pathways for PGE_2 _is presented**. The control of tissue level of PGE_2 _is both through synthesis of PGE_2 _by the COX enzyme in and its export from pro-inflammatory cells as well as by the removal of PGE_2 _from the intercellular space by prostaglandin transporter, PGT, and the efficiency of catabolism of PGE_2 _by enzymes such as 15-prostaglandin dehydrogenease. For instance, expression of the COX-2 enzyme is regulated through many pathways of which several are affected in CRC. As examples of this, somatostatin, SS, has a dampening effect on COX-2 expression, while an autocrine pathway through an epidermal growth factor receptor, EGFR, an EP4 receptor, and microRNA stimulation increase the expression and/or activity of the enzyme. Furthermore, the activity in PGE_2_-signalling pathways may vary with the expression of the PGE_2 _receptor subtypes, EP1, EP2, EP3 and EP4, which is affected in CRC. Removal of PGE_2 _from the extracellular compartment around target cells is by diffusion to the blood stream and uptake and degradation in lung, liver and kidney endothelial cells. Different cellular signalling pathways for PGE_2 _operation are indicated in the target cell. The activity in various short-term and long-term pathways, as indicated in the target cell, is increased with the CRN/CRC conditions and therefore affecting a host of cell responses, including ion secretion. "Various responses" as mentioned in the figure refers to differentiation, proliferation, survival/apoptosis, exocrine secretion, altered immune response, invasiveness/metastasis, angiogenesis. Abbreviations: AA = arachidonic acid, CBR = carbonyl reductase - also involved in degradation of PGE_2_, COX1/2 = cyclooxygenase isoforms 1 and 2, EGFR = epidermal growth factor receptor, GPCR = G protein-coupled receptors, miRNA = microRNA, MRP4 = multi-drug resistance related polypeptide 4 - example of an ABC export pump, 15-PGDH = 15-prostaglandin dehydrogenase, PGT = prostaglandin transporter, PL = phospholipid, PLA_2 _= phospholipase A_2_, RTK = tyrosine kinase receptor pathway, SS = somatostatin, sst3/5 = somatostatin subtype receptor 3 or 5.

By their inhibitory action on the cyclooxygenase enzyme (COX), non-steroidal anti-inflammatory drugs (NSAIDs) are partly chemopreventive against CRC, an effect maybe particularly due to attenuation of the enzyme isoform 2 (COX-2) [3-6]. This protection is believed to be mediated, at least in part, through reduction of prostaglandin E_2 _(PGE_2_) levels [7,8], as PGE_2 _promotes cell growth, migration and angiogenesis and reduce apoptosis [4]. In general, the downstream cascades of the PGE_2 _signaling are altered (due to gene mutations) in CRN [9-11]. These mutations may both affect PGE_2 _production *per se *via a regulation of COX enzymes in pro-inflammatory cells including epithelial cells as well as the PGE_2_-dependent signaling pathways in target cells, Figure 1. Whether changes in levels of PGE_2 _are primary or secondary causes of CRN remains unclear. With respect to PGE_2 _receptors, CRC cells and their neighboring cells have augmented expression of receptors EP2 and EP4, while initially the EP3 receptor expression often is lowered [4,8,12-15], Figure 1.

PGE_2 _is the primary endogenous agonist for the EP receptors and stimulates all 4 EP receptor subtypes [16]. Butaprost is a selective EP2 agonist [16]. Sulprostone is mainly an EP3 agonist but it is also a weak agonist of EP1 [16]. PGE_1 _(OH) is mainly an EP4 agonist but it is also a weak agonist of EP3 [16].

Studies of transport mechanisms in human intestinal epithelia in vivo require invasive bothersome procedures. In order to circumvent these problems, the Modified Ussing Air-Suction (MUAS) chamber has been developed for the study of duodenal and colonic epithelia in vitro [17,18]. Fairly easily, this method enables us to study epithelial electrogenic ion transport in human biopsies obtained during endoscopy and has been proven useful for functional receptor studies [19].

In this study we sought to establish possible differences in functional expression/response of PGE_2 _EP-receptor subtypes in colonic biopsies from CRN patients and control subjects by the use of indomethacin, PGE_2 _and selective EP receptor agonists. Our findings indicate that normally appearing colon in CRN patients, including CRC patients, express higher COX enzyme activity than in control patients but with no difference in the functional expression of the four EP receptor subtypes with respect to transepithelial ion transport.

## Methods

### Study population

Patients referred for colonoscopy were asked to participate. Patients agreeing were pooled into the neoplasia group (i.e. N-patients) if they presented a history of CRN or if CRN was macroscopically detected during colonoscopy. Patients with no previous history or present endoscopic signs of CRN served as controls (i.e. C-patients). Patients with haemorrhagic diathesis, inflammatory bowel disease or previous sigmoid resection were excluded from the study. A total of 63 patients were enrolled, hereof 45 C-patients and 18 N-patients. Among the C-patients the mean age was 59 years and the fraction of men was 41%. In the N-patients group the mean age was 55 years and 32% were men. We noted patients' medication, body mass index, previous illness, all signs of earlier colorectal disease and the findings from the colonoscopy at the time of examination. During colonoscopy, biopsy specimens were obtained from each patient. Biopsies were pinched from normally appearing mucosa, 30 cm orally from the anus on retraction of the endoscope. Standard biopsy forceps (Boston Scientific, Radial Jaw 3, outside diameter of 2.2 mm) were used. Biopsies were placed in iced Ringer-solution and immediately transferred to the laboratory for mounting in MUAS chambers.

### Ethics

The study protocol was approved by the Scientific Ethical Committee for Copenhagen (KA 97161) and Frederiksberg Counties (KF01-232/03) and conducted in accordance with the Helsinki declaration. All patients participating gave written informed consent.

### Mounting of biopsies and electrical measurements

**Figure 2:**
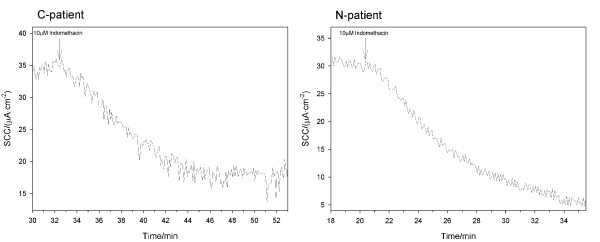
**Typical traces of the tissue response to inhibition with 10 μM indomethacin**.

Biopsies were mounted within 30 minutes in the validated MUAS chambers [18]. Mounting was carried out at 10 times magnification by means of a stereomicroscope to secure mucosa-serosal orientation and proper fixation. The exposed tissue area was 3.4 mm^2^. Both sides of the tissue were bathed in bicarbonate-Ringer solution containing the following in mM: 140 Na^+^, 4 K^+^, 121 Cl^-^, 1 Ca^2+^, 0.5 Mg^2+^, 0.5 SO_4_^2-^, 25 HCO_3_^-^, and oxygenated with 95% O_2_/5% CO_2_, circulated by gas-lift. Media were further added 11 mM D-glucose at the serosal side and 11 mM D-sorbitol at the mucosal side. Temperature was maintained at 37°C by water jackets. Short circuit current (SCC, μA·cm^-2^) and slope conductance (G, mS·cm^-2^) were recorded continuously using an automated voltage-clamp device [17]. Correction for the resistance in solutions was performed immediately before each new specimen was mounted. Our technique of fixing colonic biopsies with air-suction through a 40 μm width suction-sleeve results in fairly high slope conductance most likely due to an edge-leak around the rim of the disk aperture. Conversely, the fixing method reduces potential edge damage of the tissue [17,18]. Problems that are related to an edge-leak may be minimized, by performing an accurate solution correction just prior to insertion of the biopsy. Thus, in comparison with other studies on human colonic biopsies [20] the present technique has a high edge-leak conductance but with reduced edge damage. Therefore, our suction system is very sensitive to minute interferences as indicated by the "noise" in the SCC signals in Figure 2. Meanwhile, on the premises of correct solution correction and voltage clamping, the absolute values of drug-induced differential changes in SCC should be valid and allow quantitative comparison from tissue to tissue.

Experiments were performed after an equilibration period of 15 minutes. Various stimulators (forskolin or theophylline) and inhibitors (indomethacin, bumetanide or ouabain) of epithelial ion transport, as well as EP receptor agonists (PGE_2_, PGE_1_-(OH), butaprost or sulprostone) were then added separately to the serosal bathing solution, except for indomethacin, which was added to both sides. Indomethacin was always added 15 minutes prior to the addition of EP receptor agonists in order to minimize tissue prostaglandin production. Choices of concentration for the various drugs were based on experience from previous studies [18,21]. Bumetanide was added after EP agonist experiments on the biopsies had been completed. Forskolin or ouabain was added at the end of the experiment as a control of biopsy viability.

### Compounds

All drugs were purchased from Sigma (Vallensbaek Strand, Denmark) except for bumetanide, which was a gift from Leo Pharmaceuticals, Denmark.

### Medication and co-morbidity

At the time of the examination, one patient was medicated with corticosteroids, two with laxatives, three with selective serotonin reuptake inhibitors and other three with NSAIDs, all on a daily basis. Two N-patients had previous resections of the transverse or ascending colon for neoplasia. Results for medicated patients fell within the range of other patients (data not shown).

### Data and statistical analysis

Data are presented as mean ± SEM (number of biopsy specimens, number of patients). Wilcoxon Rank-sum test or unpaired t-test was used for the calculation of the p-values, depending on the results of the normality test and the equal variance test. P-values less than 5% were considered significant. All statistics was done on SigmaStat 2.03 for Windows, SPSS Inc., USA.

### Histological examination

Following mounting and stimulation in the MUAS chamber, biopsies considered for histological examination were fixed in 4% buffered paraformaldehyde. After embedding the tissue samples in paraffin, they were cut into 10-μm sections and stained with haematoxylin/periodic acid Schiff for examination in a Leitz Ortoplan microscope (Wetzlar, Germany). Protocols were blinded to the examiner.

## Results

### Basal levels

N-patients baseline values did not differ as compared to C-patients. The baseline SCC in the C-patients was 38.1 ± 7.6 μA·cm^-2 ^(97,36) and in N-patients 41.3 ± 15.3 μA·cm^-2 ^(39,14), p = 0.70. Baseline G in C-patients was 91.2 ± 6.3 mS·cm^-2 ^(94,36) compared to 90.4 ± 8.1 mS·cm^-2 ^(39,14) in the N-patients, p = 0.78. The slope conductance was followed through experiments to ensure stable fixation. An annotated statement on the relatively high conductance values is given in the "Methods" section.

### Stimulation

**Figure 3:**
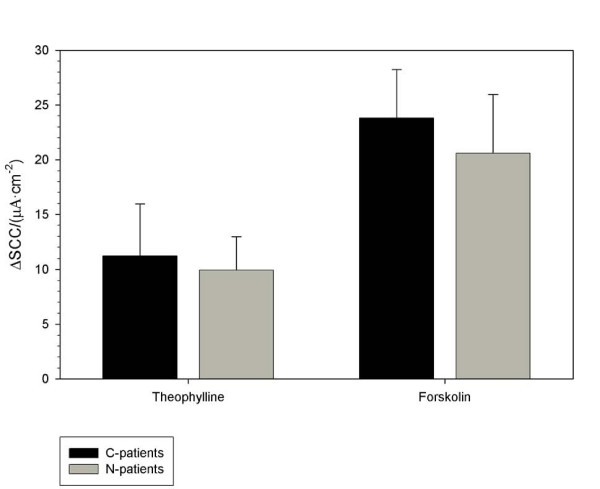
**Theophylline and forskolin-stimulated short circuit current (SCC) in human colon endoscopic biopsies from C-patients and N-patients is presented**. Numbers in parenthesis are numbers of biopsy specimens and number of patients. Increases in SCC after stimulation with 400 μM theophylline (C-patients: 29,18), (N-patients: 7,4) and 1 μM forskolin (C-patients: 45,23), (N-patients: 13,7).

**Figure 4:**
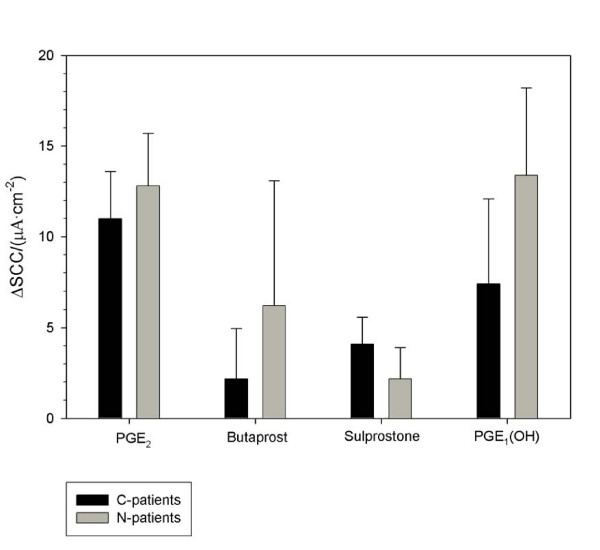
**EP receptor subtype agonists (PGE_2_, butaprost, sulprostone, and PGE_1_-(OH))-stimulated short circuit current (SCC) in human colon endoscopic biopsies from C-patients and N-patients is presented**. Numbers in parenthesis are numbers of biopsy specimens and number of patients. Increases in SCC after stimulation with 200 nM PGE_2 _(C-patients: 8,5), (N-patients: 6,3); 1.5 μM butaprost (C-patients: 12,10), (N-patients: 11,5); 1 μM sulprostone (C-patients: 11,7), (N-patients: 10,6) and 375 nM PGE_1_-(OH) (C-patients: 7,6), (N-patients: 9,4).

There were no significant differences between N-patients and C-patients in the induced SCC when stimulating with either PGE_2 _(200 nM), butaprost (1.5 μM), sulprostone (1 μM), PGE_1 _(OH) (375 nM), theophylline (400 μM) or forskolin (1 μM), Figures 3 and 4. Half-times for the SCC stimulation were equal in the two groups and lumped here for both N- and C-patients: PGE_2 _144 ± 23 s (14,8), butaprost 221 ± 11 s (23,15), PGE_1 _(OH) 191 ± 15 s (16,10), sulprostone 108 ± 15 s (21,13), theophylline 139 ± 9 s (36,22), and forskolin 304 ± 11 s (58,30).

### Inhibition

**Figure 5:**
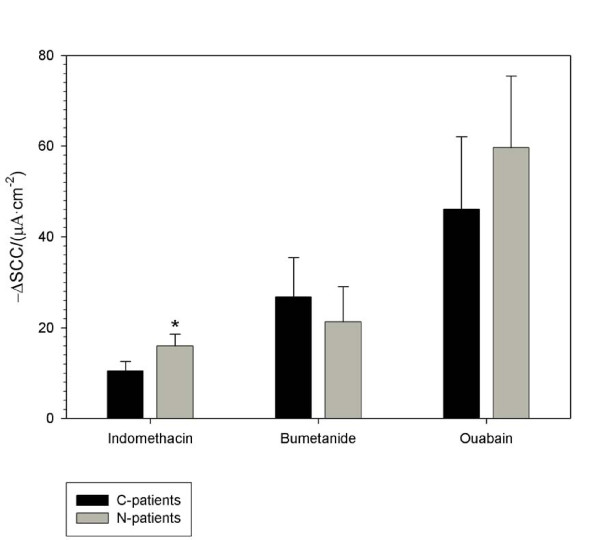
**Indomethacin, bumetanide, and ouabain-induced inhibition of short circuit current in human colon biopsies**. Inhibition of electrogenic secretion in endoscopic mucosal biopsies from the sigmoid part of colon from C-patients and N-patients. Numbers in parenthesis are numbers of biopsy specimens and numbers of patients. Decreases in short circuit current (SCC) after inhibition with 10 μM indomethacin (C-patients: 43,24), (N-patients: 21,12); 50 μM bumetanide (C-patients: 12,7), (N-patients: 8,5) and 200 nM ouabain (C-patients: 10,7), (N-patients: 8,5). * p < 0.05.

The SCC in N-patient biopsies were significantly more sensitive to indomethacin (10 μM), p = 0.027, compared to C-patients. There were no significant differences between N-patients and C-patients with respect to the inhibiting effect of bumetanide (50 μM) or ouabain (200 nM), Figure 5. Halftimes for the inhibition of SCC were equal for N- and C-patients and lumped together: for indomethacin 170 ± 11 s (64,36), for bumetanide 64 ± 8 s (20,12) and for ouabain 131 ± 13 s (18,12). While the drug-elicited changes in SCC amplitude may vary between groups of patients, the lack of difference between N- and C-patients in response halftimes for stimulation and inhibition of SCC is simply indicating that the same mechanism is affected in the biopsies by each individual drug.

### Histological examination

Histological assessments of previously mounted biopsies were performed for the extent of tissue pathology, edge damage and the thickness of biopsies. The damage found in biopsies were denoted by a severity score ranging from 0 to 4; 0 being severe mucosal damage and 4 being no mucosal damage; as previously described [18]. No difference in histology was detectable for N-patients as compared to C-patients, data not shown. In particular, no signs of inflammation or neoplasia were detected in the biopsies from N-patients. Histological examination showed good correlation between mucosa damage and electrical parameters. Thus, disrupted mucosal layers were almost always non-responding to stimulation and inhibition in the MUAS chambers. Biopsies scoring 0-1 had an average increase in SCC of 2.8 ± 2.0 μA·cm^-2 ^(53,29) in response to stimulation with forskolin, which was significantly less compared to an average of 20.5 ± 4.0 μA·cm^-2 ^(56,25) in the biopsies scoring 2-4, p < 0.001.

## Discussion

The present study provides evidence that electrogenic transport is altered in histological normal appearing colonic mucosa in CRN patients with respect to indomethacin-sensitive mechanisms. This finding supports that NSAID-sensitive mechanisms are activated not only in tumor tissue [22] but also in normal appearing tissue.

Levels of PGE_2 _in the para-/auto-crine milieu are elevated in CRN [23-25] and the clinical benefits of reducing PGE_2 _levels for CRC has been documented [5,22,26-30]. Accordingly, related therapeutic strategies are suggested [31,32]. But what are the mechanisms for the elevated PGE_2 _levels in CRN and how do we explain the effect of indomethacin in the present study?

Several enzymes and regulatory pathways control the level of PGE_2 _both in colonic cells and in the intercellular environment of colonic tissue, Figure 1. In particular, it appears that the COX-2 enzyme expression and hence its activity is elevated in human colonic carcinogenic cells [27,33-35]. Activation of COX-2 enzymes leads to an immediate increase in the intracellular level of PGE_2_, Figure 1. Regulation of the COX-2 expression is simultaneously controlled by a host of intracellular and extracellular signaling pathways [35-37], Figure 1. In addition, the extracellular concentration of PGE_2 _is controlled by several other means. Increasing the extracellular PGE_2 _through prostaglandin secretion in ABC efflux transporters, MRP4 and MOAT, is probably augmented by elevated activity in the MRP4 transporter as found in CRC cells, Figure 1[38]. Removal of extracellular PGE_2 _is partially performed by a specific prostaglandin transporter, PGT, and further degradation of PGE_2 _is by the enzymes 15-prostaglandin-dehydrogenase, 15-PGDH, and carbonyl reductase, CBR, of which, both the activity by PGT and 15-PGDH are reduced in CRC cells, Figure 1[3,38,39]. Additional support for the involvement of altered15-PGDH enzyme activity in CRC development was recently documented in a mouse model of 15-PGDH -/-mouse demonstrating resistance to COX-2-inhibitor celecoxib' prevention of colon tumors [40]. Thus, the combined effects of increased synthesis and export together with reduced elimination and degradation of extracellular PGE_2 _in CRC point to a maintained higher level of PGE_2 _in the auto-/paracrine milieu in the CRC colonic epithelium, Figure 1. Interestingly, indomethacin down-regulates the expression of some of the ABC transporters, which are up regulated in CRC [38,41]. Therefore several mechanisms could account for indomethacin in regulating the bioavailability of PGE_2_.

In the present study we found no difference in basal electrogenic transport values or in the response to stimulation with PGE_2 _or EP receptor subtype specific agonists. Thus, the observed altered expression of EP receptor subtypes in CRC patients compared to non-CRC subjects does not seem to be manifest in the functionality of the receptors in epithelial transport. We speculate that the observed increased sensitivity to indomethacin in the N-patients is due to a higher baseline production of prostaglandins in N-patients following an increased expression/activity of the COX-2 enzyme and export of PGE_2 _[27,38] and/or a lowered removal and degradation of PGE_2_, Figure 1[38,39]. This would be consistent with the lack of differences between specific agonists of the EP receptors as well as still explaining the chemopreventive effect of the NSAIDs. To prove the involvement of the isoform COX-2 as a direct link to our findings, experiments with a specific COX-2 inhibitor would be required.

Although there is no significant difference in the baseline secretion measured as SCC between N-patients and C-patients, the basal current is nearly three times higher than the indomethacin-induced lowering of the current. The significantly higher (5 to 6 μA·cm^-2^) differential inhibition of the basal current by indomethacin in N-patients compared to C-patients is not discernable as a significant difference in the basal SCC as such. Biopsies from N-patients had a mere non-significant (3.3 μA·cm^-2^) higher value of SCC in the total basal current compared to C-patients. A straight forward explanation for this lack of significance is that the total basal current is most likely a sum of several different electrogenic transport processes including amiloride-sensitive sodium absorption and Ca^2+^-induced anion secretion, not accounted for in the indometacin-sensitive transport. This is substantiated by a partial inhibition of the current by bumetanide and an even higher inhibition of the total current by ouabain compared to indomethacin inhibition, Figure 5. Thus a significant difference in a single transport type, as for indomethacin, is possibly smeared to insignificance by its inclusion in the total basal current with a higher variability.

Of the four employed EP receptor agonists, PGE_2 _induced the largest increase in SCC (i.e. electrogenic secretion), which is not surprising as PGE_2 _stimulates all four EP receptor subtypes. EP4 seems to be the receptor subtype that is responsible for the largest proportion of this secretion, as stimulation with PGE_1 _(OH) brought about the largest increase in secretion. This fits with findings in the human duodenum using the same experimental technique [19]. Similar mechanisms appear to be present in rat colon, but in contrast to rats, where EP4 is the major mediator of the PGE_2 _response [21], the human colon PGE_2_-induced secretion seems to be induced by all four receptor subtypes, EP4, EP2 and EP1/EP3, Figure 4. This underlines the notable difference of functional EP receptors in some animals as compared to man. However, in agreement with findings in rat colon [21], PGE_2 _was found to be only a partial agonist of secretion, as forskolin almost doubled the electrogenic secretory response of PGE_2_. It is not possible to make any final conclusions regarding the observed differences between human and rat colon, although species or regional characteristics might explain the observed differences.

The PGE_1_-derivative Lubiprostone is used clinically as a laxative. Besides its prokinetic effect, Lubiprostone is also believed to be a chloride channel, ClC-2, opener and now further a stimulant of EP4 receptor subtypes in human ileum and colon epithelium chloride secretion, dependent on a well-functioning cystic fibrosis transmembrane conductance regulator protein, CFTR [42], as well as of the EP4 receptor subtype in human and rat duodenal epithelial bicarbonate secretion [19,43]. This information corroborates our use of PGE_1_(OH) as a rather specific EP4 receptor subtype agonist and that this receptor subtype is the major pathway for PGE_2_-induced colonic anion secretion in man, Figure 4.

## Conclusion

Epithelial electrogenic transport is altered in histological normal appearing colonic mucosal biopsies from patients with previous or present colorectal neoplastic disease with respect to indomethacin-sensitive mechanisms. The mode of action for the observed effect of indomethacin does not seem related to the functionality of expressed prostaglandin EP receptor subtypes, EP1-4, when compared with non-neoplastic patient tissues. Therefore, our results point directly to a change in the activity of COX enzymes in the normal colonic tissue from patients with neoplasia, although at present we cannot differentiate between the isoform of COX enzymes possibly being involved. To do this selective COX-1 and COX-2 inhibitors would have to be tested.

## Abbreviations

COX: cyclooxygenase; COX-2: cyclooxygenase isoform 2; C-patients: control-patients; CRC: colorectal cancer; CRN: colorectal neoplasia; G: slope conductance; MUAS: Modified Ussing Air-Suction; NSAIDs: non-steroidal anti-inflammatory drugs; N-patients: neoplasia-patients; PGE_2_: prostaglandin E_2_; SCC: short circuit current; SEM: standard error of the mean.

## Competing interests

The authors declare no competing interests. After completion of the study Mark Berner Hansen was employed at AstraZeneca Research & Development.

## Authors' contributions

NK carried out Ussing chamber studies, participated in the design of the study, performed statistical analysis and helped to draft the manuscript. CT and PSO carried out Ussing chamber studies and participated in the design of the study. ABW carried out Ussing chamber studies, performed statistical analysis and helped draft the manuscript. SSP carried out histoanatomical studies. MBH and NB conceived the study and helped draft the manuscript. All authors read and approved the final manuscript.

## Pre-publication history

The pre-publication history for this paper can be accessed here:

http://www.biomedcentral.com/1471-230X/10/9/prepub
